# Melanin Stacking Differences in Pigmented and Non-Pigmented Melanomas: Quantitative Differentiation between Pigmented and Non-Pigmented Melanomas Based on Light-Scattering Properties

**DOI:** 10.3390/life13041004

**Published:** 2023-04-13

**Authors:** Frederick H. Silver, Tanmay Deshmukh, Hari Nadiminti, Isabella Tan

**Affiliations:** 1 Department of Pathology and Laboratory Medicine, Robert Wood Johnson Medical School, Rutgers, The State University of New Jersey, Piscataway, NJ 08854, USA; 2 OptoVibronex, LLC, Bethlehem, PA 18015, USA; 3 Summit Health, Dermatology Department, Berkeley Heights, NJ 07922, USA

**Keywords:** skin cancer, basal cell carcinoma, squamous cell carcinoma, melanoma, fibrosis, blood vessels, VOCT, OCT, pigmented melanomas, non-pigmented melanomas, melanin stacking, light scattering

## Abstract

Cutaneous melanoma is a cancer with metastatic potential characterized by varying amounts of pigment-producing melanocytes, and it is one of the most aggressive and fatal forms of skin malignancy, with several hundreds of thousands of cases each year. Early detection and therapy can lead to decreased morbidity and decreased cost of therapy. In the clinic, this often translates to annual skin screenings, especially for high-risk patients, and generous use of the ABCDE (asymmetry, border irregularity, color, diameter, evolving) criteria. We have used a new technique termed vibrational optical coherence tomography (VOCT) to non-invasively differentiate between pigmented and non-pigmented melanomas in a pilot study. The VOCT results reported in this study indicate that both pigmented and non-pigmented melanomas have similar characteristics, including new 80, 130, and 250 Hz peaks. Pigmented melanomas have larger 80 Hz peaks and smaller 250 Hz peaks than non-pigmented cancers. The 80 and 250 Hz peaks can be used to quantitative characterize differences between different melanomas. In addition, infrared light penetration depths indicated that melanin in pigmented melanomas has higher packing densities than in non-pigmented lesions. Using machine learning techniques, the sensitivity and specificity of differentiating skin cancers from normal skin are shown to range from about 78% to over 90% in this pilot study. It is proposed that using AI on both lesion histopathology and mechanovibrational peak heights may provide even higher specificity and sensitivity for differentiating the metastatic potential of different melanocytic lesions.

## 1. Introduction

Cutaneous melanoma is a cancer with metastatic potential characterized by varying amounts of pigment-producing melanocytes displaying new friable vessels and stiff fibrotic extracellular matrix [1]. It is one of the most aggressive and fatal forms of skin malignancy, with 351,880 new cases in 2015 [2]. There is great importance in diagnosing melanoma in its early stage since prognosis is directly proportional to the severity of the neoplasm [3]. Early detection and therapy can lead to decreased morbidity and decreased cost of therapy [4]. In the clinic, this often translates into annual skin screenings, especially for high-risk patients, and the use of the ABCDE evaluation of lesions (asymmetry, border irregularity, color, diameter, evolving) criteria [4]. There are other methods clinicians use to classify melanomas, much of which evolves from their clinical experience and expertise in the discipline [3].

Several classifications of melanomas have been proposed for a tumor: (1) 0.8 mm or smaller; or (2) greater than 0.8 mm; (3) with and without ulceration; and (4) if it has spread to one or more lymph nodes close to the initial disease site [5]. However, the use of these classifications requires information from an excisional biopsy as opposed to being based on non-invasive diagnoses using visual inspection and dermoscopy.

As a result, much effort has been made and continues to be expended in developing and refining effective diagnostic algorithms to help identify melanomas and differentiate them from nevi using asymmetry, irregularity, color variation, and size and shape [6]. However, it would save clinical time and improve patient satisfaction to have a rapid method to screen skin lesions using a non-invasive technique.

Melanoma is the deadliest form of skin cancer. In the early stages, melanoma can be successfully treated with surgery; however, upon metastasis, the rates of survival drop drastically. Thus, early detection and accurate diagnosis are necessary for lowering mortality and ensuring the best prognosis for patients. The diagnosis of melanoma anecdotally relies on the generous use of dermoscopy and biopsy for suspicious lesions by dermatologists, surgeons, and other clinicians. Although these traditional methods are cornerstones in clinical practice, there are emerging techniques that can improve the diagnostic sensitivities and specificities of early melanoma, optimizing lesion selection for biopsy and histopathological examination. These can give rise to early triaging to avoid unnecessary surgery and biopsy while simultaneously improving clinical outcomes. In addition to these advances, early recognition, detection, and treatment of melanoma remain essential. Melanoma’s unique presentation and identification through traditional dermoscopy, computer-aided image analysis, and, most recently, machine learning and artificial intelligence (AI) methods require a detailed analysis. Trends in the current literature dictate that there is a need for more quantitative staging, which may be achievable through the integration of AI and machine learning diagnostics. As current approaches are improved upon and as novel technologies are developed, the refined diagnostic ability will strive toward the ultimate goal of reducing mortality by melanoma.

Melanoma is an important concern in public health, both in the United States and worldwide. Incidence rates in the United States have doubled between 1982 and 2011, and the annual cost of treating newly diagnosed melanomas is forecasted to triple by 2030 [7]. Sun-protective behaviors, including sunscreen application and wearing sun-protective clothing, can reduce skin cell damage due to exposure to harmful ultraviolet (UV) radiation.

Melanoma also has many faces, which often obscures the initial identification of atypical lesions for further investigation [7]. There is great importance in diagnosing melanoma in its early evolution since the prognosis is directly proportional to the severity of the neoplasm [6,7]. Early detection and treatment can lead to decreased morbidity and decreased cost of therapy [8]. The routine method to evaluate an atypical lesion is biopsy, followed by histopathological examination [8]. The challenge lies in identifying suspicious lesions at the earliest time possible in their evolution. Recent research reflects the need to focus on quantifiable markers and non-invasive methods for early melanoma diagnosis in hopes of avoiding unnecessary biopsy interventions, thus preventing associated complications such as infection and scarring [9]. These analyses also support the application of precision medicine to melanoma, as the literature suggests the future of the early detection of melanoma lies in a multi-pronged approach, which includes targeted surveillance of high-risk patients using imaging techniques with the integration of artificial intelligence systems [9,10,11]. This research is of utmost importance and relevance as melanoma and other skin cancers remain of high incidence and mortality in the United States and worldwide.

### 1.1. Central Features in Early Diagnosis

Within the past 30 years, the diagnosis and recognition of early melanoma have evolved. In the 1990s, dermoscopy was the main tool for examining cutaneous subsurface features [7,8]. In the 2000s and up until now, newer techniques using computer-aided analyses and digital diagnostics are emerging technologies to supplement the clinician’s eye [10,11]. There are also significant differences in diagnoses based on whether the lesion is pigmented or non-pigmented [11].

Non-pigmented or “amelanotic” melanomas can present in a range of colors, from pink or red to purple or colorless [3]. These lesions are often diagnosed using dermoscopy, with a close examination of vascular patterns; however, it is difficult to visualize vessels with sufficient precision solely using dermoscopy [7]. Thus, there is a need for an alternative, non-invasive technique that would allow for the examination of deeper subsurface vessel architecture.

In 1985, an acronym termed “ABCD” (asymmetry, border irregularity, color variation, diameter >6 mm) was devised to educate physicians and the public to recognize the early clinical presentation of pigmented melanoma [3,12]. However, based on clinical experience, these features were found to be most strongly associated with lesions greater than 6 mm [3]. Thus, these guidelines are useful in diagnosing a subset of melanomas: early, thin neoplasms that are otherwise mistaken for benign pigmented lesions [8].

Atypical melanocytic lesions are benign, pigmented lesions that contain abnormal cells but have the potential to progress into a melanoma [11]. These lesions are often difficult to differentiate from melanoma; however, melanoma can have distinct features, including ulceration, depth for staging, and texture [3]. This is a significant limitation as color characteristics and staging are subjective and qualitative in nature. In the clinic, these lesions are typically examined using the ABCD criteria, then excised and sent for histopathological examination to determine the definite diagnosis [3,12]. Thus, quantifiable and non-invasive tools are necessary to avoid unnecessary excision and associated complications such as infection and cosmetic scarring [9].

Lesion evolution is also a critical feature in cutaneous melanoma and has led to the advancement of the initial ABCD criteria to include “E” for “evolving” [3,12]. This enhancement is of special importance in diagnosing nodular lesions, which often present at more advanced stages as smaller lesions [3,12]. The ABCDE tool is a straightforward, memorable method that has educated the public, non-dermatologists, and dermatologists alike on the cardinal features of melanoma.

### 1.2. Dermoscopy

Traditional dermoscopy uses a lighted magnifier as a hand-held device, allowing for quick visual analysis of subsurface features of skin lesions [13]. These lesions and structures are first identified by the clinician’s unaided eye and often include networks, streaks, or veils that are characteristic of melanoma [13]. Dermoscopes usually result in 10-fold magnification of the skin, which greatly improves the visualization of epidermal and papillary dermal features [3]. A systematic review revealed that there was an increase in sensitivity and specificity from 71% to 90% when using dermoscopy as opposed to the unaided eye [3]. However, the efficacy of this tool requires experience, namely, in the first step of identifying lesions to be further investigated. After dermoscopy, a decision tree often follows, in which the clinician evaluates the lesion for potential biopsy [3]. If deemed potentially melanocytic, there is a scoring system that allows for the classification of the lesion as benign, suspicious, or malignant [7,9]. There are a few hallmarks that distinguish a malignant lesion from a benign pigmented lesion, which include atypical pigmented networks, streaks, globules, asymmetry, and blue-white features [8,9].

Dermoscopy is often used in conjunction with other clinical measures, including the ABCDE criteria and pattern analysis, along with the examination of color, architecture, homogeneity, and symmetry [3,13]. Still, the efficacy of these tools requires clinician expertise, especially in initial identification [3]. There are also notable handicaps associated with dermoscopy, one being its potential inability to detect very early and “featureless” melanomas [3,14].

### 1.3. Computer-Augmented Image Analysis

Computer-assisted melanoma diagnosis was first introduced in the 1990s [15]. Some of the most widely used computer-augmented tools include assisted dermoscopes, which use computer software to overlay with a previous image, which is especially helpful in a follow-up setting [15]. There are also methods that use total-body photography, creating a digital map that picks up on certain pigmented lesions to be further examined [16]. Other tools utilize empirical databases for comparison, along with fiber-optic imaging and ultrasound technology [2,3].

The basis of these methods is often image capture, which can be used in follow-up skin examinations as baseline comparisons in skin screenings or when suspicious changes are identified [17,18,19]. However, there is no clear method offering complete accuracy in melanoma diagnosis, and clinician expertise is still essential.

### 1.4. Digital Dermoscopy, AI, and Machine Learning

Computerized methods have recently been implemented in clinical practice as assistive tools in making melanoma diagnoses [17]. These tools allow for the better quantification of differences between melanoma and other atypical cutaneous lesions rather than relying on qualitative measures that vary based on the clinician’s determination [18]. Digital dermoscopy has improved the sensitivities and specificities of selecting lesions for biopsy since the computerized system can obtain additional data to what is seen by the naked eye and under dermoscopy [3,17].

Multispectral digital dermoscopy and image analysis is a subset of digital dermoscopy based on the increased depth penetration of light related to its wavelength [3,19]. Images are captured at varying wavelength bands, and the data obtained from different depths are used for computer analysis and comparison against a network of historical images [20]. The computer then classifies the lesion and can indicate a biopsy recommendation [20,21]. This tool allows for rapid analysis of deeper cutaneous features, some of which explore up to 2 mm below the skin’s surface [22].

There are also laser-based tools that are non-invasive ways to examine and image lesions in vivo and in real-time [21]. Some of these methods are at a high enough resolution to allow the visualization of epidermal and papillary dermal architecture without performing a biopsy [21]. This is a major focus of current research, and novel techniques are constantly being developed.

There has also been recent work on the integration of digital methods with AI networks [2,23]. For instance, the multispectral dermoscopy method has recently been modified to integrate artificial neural systems to improve its diagnostic ability [3,19]. These are more objective tools that can better control inter-operator variability, thus serving as excellent screening techniques [20,21]. The use of AI in medicine is continuously growing and, if embraced by clinicians, can strongly augment clinical decision-making and also increase access to care [23,24]. The integration of AI and machine learning in melanoma detection has the strong potential to foster a more ordered, quantitative, and non-invasive approach to recognizing and diagnosing atypical lesions [24,25].

### 1.5. Differentiation between Pigmented Lesions

The differentiation of cutaneous lesions is important, especially when discerning pigmented basal cell carcinoma (BCC) and melanoma. BCC and melanoma arise from different locations within the epidermis and can have different visual characteristics, which can sometimes be discerned by the unaided eye [26]. BCC originates in the basal cell layer of the epidermis; however, melanoma arises from malignancy in the melanocytes within the deepest part of the epidermis and typically presents as a pigmented mole [27]. The distinct locations of these malignancies often correlate with their unique presentations. However, pigmented BCC and melanoma can be more difficult to differentiate, and often, dermoscopy is used for more detailed examination [6]. Under dermoscopy, pigmented BCC is characterized by arborizing vessels, blue/grey ovoid nests, and leaf-like regions of the lesion [26]. However, melanoma under dermoscopy presents blue/white veils, atypical vascular patterns, and irregular globules [27,28], and thus, excisional biopsy is performed to obtain a definite diagnosis [7,8]. Current research focuses on improving the ease and accuracy of diagnostic tools for identifying melanomas, some of which rely on computerized methods, artificial intelligence (AI), and machine learning. Still, there is a need for further research to develop additional diagnostic tools to enhance the differentiation and diagnosis of pigmented BCC and melanoma [26].

### 1.6. New Diagnostic Technologies Including Vibrational Optical Coherence Tomography (VOCT)

Novel technologies are being developed, including computer-augmented image analysis, AI, and machine learning, to effectively screen for early melanoma [29]. The integration of these methods can improve the sensitivities and specificities of current diagnostic tools, thus optimizing lesion selection for biopsy and histopathological examination [29]. These methods can give rise to early triaging to avoid unnecessary surgery and biopsy while simultaneously improving clinical outcomes. By using AI and machine learning, diagnostic tools can capture images and data from the lesion, which can be used for computer analysis and comparison against a network of historical images [23]. Non-invasive and quantitative methods are currently under development; they utilize lasers and sound waves to determine the depth and subsurface architecture of the lesion, which can differentiate melanoma from nevi [29]. These tools have the potential to greatly improve visual diagnosis and dermoscopy as they allow for the rapid and standardized analysis of deeper cutaneous features, which can then inform the targeted need for biopsy and surgery [8].

A new method termed vibrational optical coherence tomography (VOCT) has been developed to non-invasively characterize the type and margins of skin cancers [1,8,30,31,32]. VOCT provides a quantitative vibrational spectrum that can be used to identify cellular components, blood vessels, dermal collagen, and fibrosis. New peaks associated with cancerous cells, new friable blood vessels, and cancer-associated fibrosis have been identified [1,8,30,31,32]. Fingerprints of actinic keratosis (AK), basal cell carcinoma (BCC), squamous cell carcinoma (SCC), and melanoma were shown to be significantly different from each other at a 0.95 confidence level [1]. The purpose of this paper is to test the hypothesis that “pigmented” and “non-pigmented” melanomas can be quantitatively differentiated from each other using VOCT.

## 2. Methods

### 2.1. Subjects

Normal skin (*n* = 80) was studied in vivo and in excised skin cancer biopsies (*n* = 100) in vitro using VOCT after informed consent was obtained, as reported previously [1,8,30,31,32]. The control subjects studied ranged in age from 21 to 71 years old. Tissue biopsies were studied from patients undergoing excisional therapies with a melanoma diagnosis, as described previously [1,8,30,31,32].

Pigmented (*n* = 18) and non-pigmented (*n* = 55) melanomas were studied after excisional biopsies were collected. Routine dermatological and pathologic examinations resulted in a diagnosis of melanoma. Mechanovibrational data on normal skin, melanomas, basal cell carcinomas (BCCs), and squamous cell carcinomas (SCCs) were taken from a recently published paper [1].

### 2.2. Measurement of Resonant Frequency

Measurements on normal skin in vivo and on melanoma excisional biopsies in vitro were made using an OQ Labscope 2.0 instrument modified with a 2-inch-diameter speaker placed about 2.5 inches from the tissue to be studied, as described previously [1,8,30,31,32]. Raw image data were collected using the Labscope instrument [1,8,11,12,13,14,15,16,17,18,19,20,21,22,23,24,25,26,27,28,29,30,31,32]. The measured resonant frequencies were converted into elastic modulus values using a calibration equation (Equation (1)) [1,8,30,31,32]. A normalized weighted displacement value was generated, as discussed previously [1,8,30,31,32]. Sample component displacements are inversely related to the modulus (E) in MPa of the tissue elements, where fn is the resonant frequency and d is the sample thickness in m.

E × d = 0.0651 × (fn)^2^ + 233.16 (1)

Histopathology was conducted by a dermatopathologist after routine processing. Histopathology was compared to the distribution of resonant frequency peaks, as described previously [1]

### 2.3. Machine Learning Analysis

Different machine learning algorithms were implemented and compared. The accuracy of the machine learning algorithms was optimized using a logistic regression model, as described previously [1]. Three distinct datasets were inputted into the algorithm. The datasets used were for different melanomas, where each melanoma was compared to normal skin (controls). Using these measurements, the sensitivity and specificity were calculated based on Teventhan [33] and compared to machine learning results for basal cell carcinoma and squamous cell carcinoma.

### 2.4. Statistics

The resonant frequencies of normalized peak heights of normal skin and pigmented and non-pigmented melanoma were compared using an unpaired one-tailed Student’s *t*-test. All *p*-values were considered significant if they were less than 0.05.

## 3. Results

**Figure 1:**
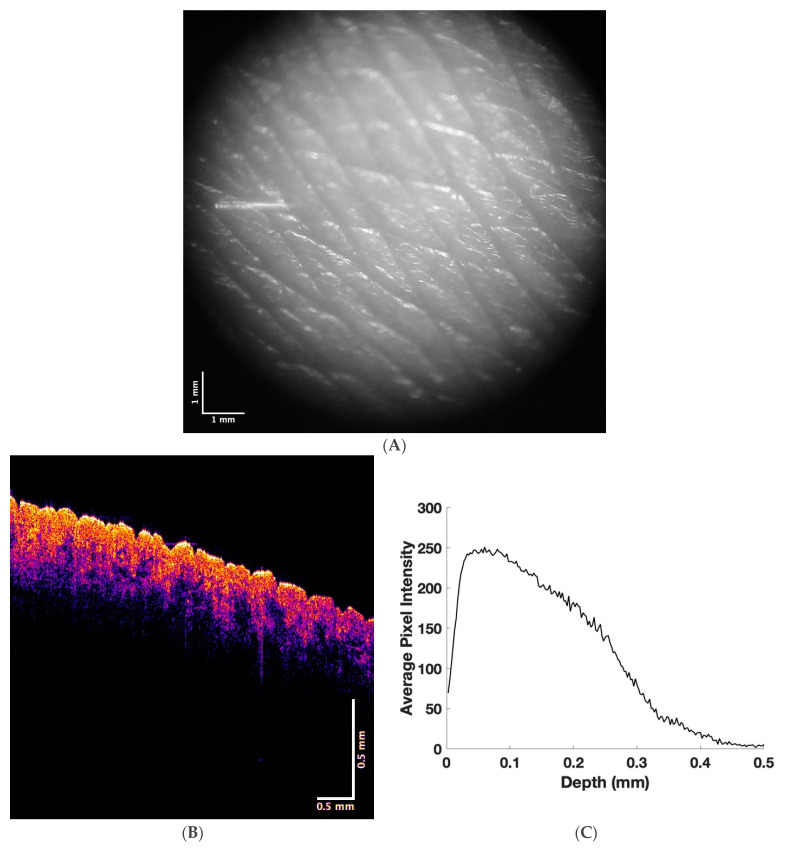
Camera (**A**) and color-coded OCT images of normal skin (**B**) and a plot of pixel intensity versus skin depth (**C**), as determined from a scan of the OCT image.

Figure 1 shows a camera image (A) of normal skin along with a color-coded OCT (B) image. Note that the color-coded OCT image shows the stratum corneum in bright yellow and the layers between the basal epithelium and stratum corneum in yellow and pink. The papillary collagen layer is shown in blue. A plot of pixel intensity versus sample depth is shown in C, determined from a scan of the OCT image. Note that the pixel intensity decreases almost linearly throughout the normal skin once the light reaches the depth of the stratum corneum.

**Figure 2:**
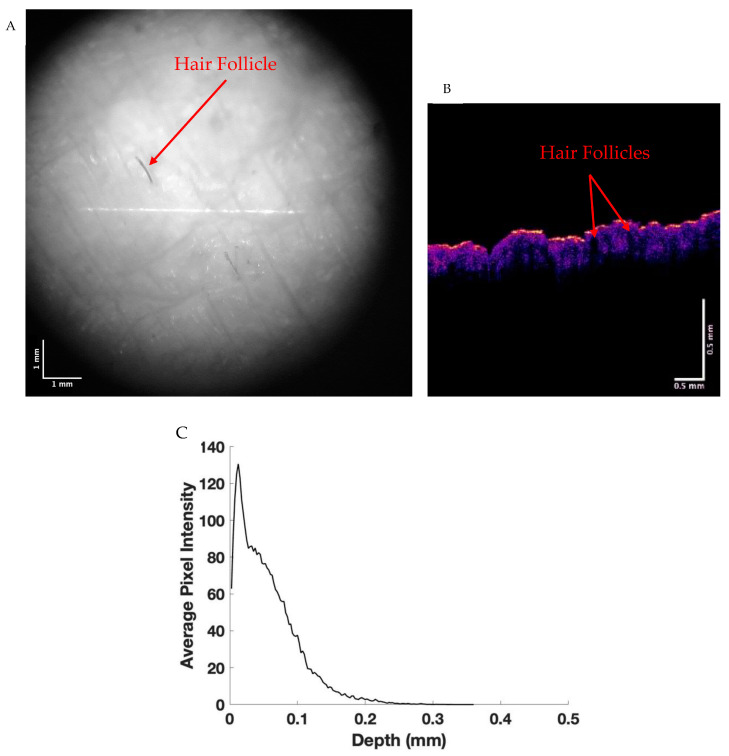
Camera (**A**) and color-coded OCT (**B**) images of a non-pigmented melanoma and a plot of pixel intensity versus depth (**C**) determined from a scan of the OCT image. Note the decreased pixel intensity versus depth compared to normal skin in Figure 1C.

In contrast, Figure 2 shows camera (A) and color-coded OCT images (B) of a “non-pigmented” melanoma. Note in A the presence of a hair follicle. A plot shows pixel intensity versus depth for a non-pigmented melanoma (C). Note that the pixel intensity versus depth is much lower than that observed for normal skin (Figure 1C) and drops very rapidly after the light penetrates the surface of the lesion. The low pixel intensity of the “non-pigmented” melanoma indicates that the melanin in the lesion scatters additional light compared to the melanin in normal skin. At a wavelength of 840 nm, melanin does not absorb infrared light [26], so the difference must be a result of light scattering and not light absorption.

**Figure 3:**
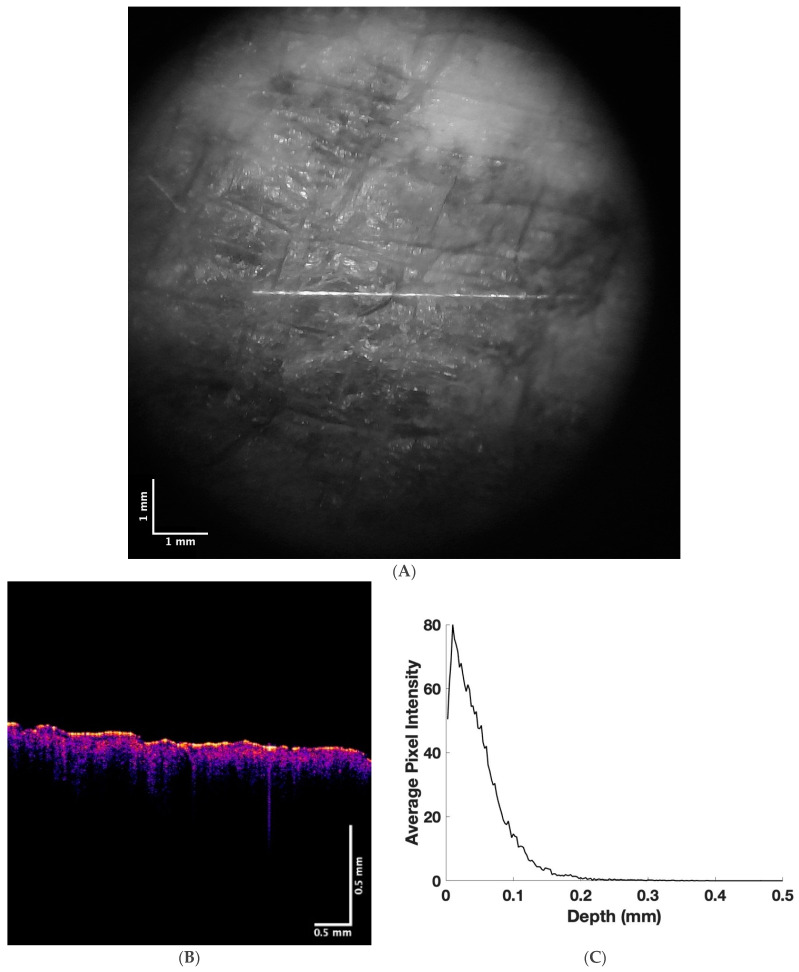
Camera (**A**) and color-coded OCT (**B**) images of a pigmented melanoma and a plot of pixel intensity versus depth (**C**) determined from a scan of the OCT image. Note the decreased pixel intensity versus depth compared to normal skin (Figure 1C) and non-pigmented melanoma (Figure 2C).

In comparison, a “pigmented” melanoma is shown in Figure 3, while the camera image (Figure 3A) clearly shows a large dark-pigmented region. The color-coded OCT image (B) of the pigmented melanoma does not look significantly different from that of the “non-pigmented” melanoma since both have lesion areas denoted by black spots. A plot of pixel intensity versus depth for the pigmented melanoma is shown in Figure 3C. Note that the pixel intensity of the surface of the pigmented melanoma is lower than that of the normal skin and the non-pigmented melanoma, suggesting that the melanin particles have different stacking densities in the pigmented melanoma than the non-pigmented melanoma. Previous studies have modeled how the size and shape of melanoma particles influence their optical properties [34,35,36].

**Figure 4:**
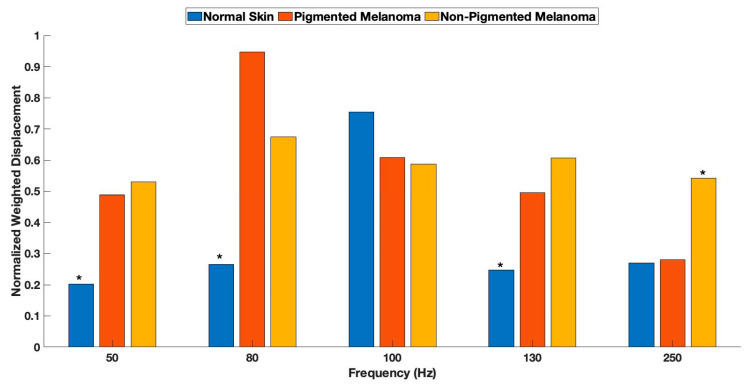
Normalized weighted displacement data obtained from VOCT measurements on normal skin, non-pigmented melanoma, and melanoma. The statistical significance of the weighted displacement at different frequencies is listed in Table 1. Asterisks on bars note statistical differences between peak heights.

Figure 4 is normalized weighted displacement data obtained from VOCT measurements on normal skin, non-pigmented melanomas, and pigmented melanomas. The statistical significance of the weighted displacement at different frequencies is listed in Table 1. Note that the differences between normal skin and melanomas are a result of differences in the 50, 80, 130, and 250 Hz peaks, as reported previously [1,14].

Table 1 lists the statistical comparisons between the resonant frequency peak heights for normal skin, non-pigmented melanoma, and pigmented melanoma. The data shown indicates that non-pigmented and pigmented melanomas can be differentiated from normal skin based on the 80 and 250 Hz peak heights. Table 2 lists a statistical comparison between the pixel intensity for non-pigmented and pigmented melanomas. The pixel depth at which pigmented lesions scatter the infrared light, preventing it from returning to the detector, is less than that observed for non-pigmented lesions, suggesting that the melanin packing density is greater in pigmented versus non-pigmented melanomas. Table 3 shows differences in sensitivity and specificity calculated from mechanovibrational data and machine learning techniques. VOCT data can be used to differentiate between different types of skin cancers non-invasively, with between 78% and 92% accuracy.

## 4. Discussion

The incidence of melanoma is increasing annually in the US and the world. In Europe, the incidence rate is 10–25 new melanoma cases per 100,000 inhabitants; in the USA, it is 20–30 per 100,000, and in Australia, it is 50–60 per 100,000 [37]. In recent years, there has been a dramatic increase in melanomas.

With 178,560 cases of melanoma diagnosed in 2018 in the United States, resulting in 9320 deaths, this disease demands more attention [37]. Some consensus reviews suggest that thin melanomas, up to 0.8 mm in tumor thickness, do not require further imaging diagnostics [37]. However, the incidence of melanoma is increasing worldwide, especially in light-skinned people that have excessive sun exposure, suggesting that more attention to development of new screening techniques may be useful. In this paper, we report that non-pigmented and pigmented melanomas are similar in their mechanovibrational spectrum peaks, while they appear to differ in their light-scattering properties. This may reflect differences in the stacking of melanin particles in the lesion.

Most histological diagnoses involving melanocytic lesions can be made with a high level of certainty; however, there exists a subset of melanocytic neoplasms that can be difficult to classify as benign or malignant based on conventional microscopic analysis. These lesions are often referred to as atypical melanocytic proliferations [38]. Today, many primary melanomas have a diameter of less than 5 mm, and early diagnosis of these cancers is important. It may be important to further characterize the light-scattering properties of different melanomas to discern if “non-pigmented” melanomas have smaller pigment aggregates. In this study, we report that the light-scattering properties of pigmented and non-pigmented melanomas appear to be different, suggesting that evaluation of melanin particle stacking may be an important diagnostic clue to the early diagnosis of non-pigmented melanomas.

Although uncommon, malignant melanoma (MM) accounts for less than 2% of all melanomas; early diagnosis of MMs is of vital importance for appropriate management and a successful outcome. The inability to recognize unusual MM variants is a real challenge for clinicians and pathologists and has a critical impact on patients [38]. The size, shape, and melanin aggregate content may provide clues to differentiate benign lesions from potential metastatic cancers. However, other non-invasive techniques besides dermoscopy and visual inspection may be needed to diagnose the melanin content of a skin lesion effectively and non-invasively.

When used by specialists, dermoscopy is better at diagnosing melanoma compared to the inspection of suspicious skin lesions by eye. Dermoscopy use is prevalent among dermatologists; with appropriate training, dermoscopy may also be used by primary care physicians who perform skin examinations for the purpose of detecting skin cancer [39]. Dermoscopy is more accurate when interpreted with the patient present rather than using dermoscopy images [40]. It allows an early diagnosis of melanoma, increasing the percentage of melanomas that can be detected at a diameter less than 6 mm [41]. However, it may be necessary to measure lesion light-scattering properties non-invasively to make more definitive conclusions about the presence of a non-pigmented melanoma before it grows to a diameter and depth of more than 0.5 mm.

In reviewing the literature, different histological forms of melanomas have been identified based on color, shape, type of blood vessel, and other descriptive methods; however, these measures are difficult to use to quantitatively characterize skin cancers. Quantitative characterization of skin cancers would lead to more precise staging and more precise analysis using machine learning and artificial intelligence (AI). In addition, using AI conducted on both lesion histopathology and mechanovibrational peak heights may provide even higher specificity and sensitivity for skin cancer diagnosis and staging.

While both types of melanomas have similar characteristics, including new 80, 130, and 250 Hz peaks, pigmented melanomas have larger 80 Hz and smaller 250 Hz peaks, suggesting differences in melanin pigment particle type and size. The 80 and 250 Hz peaks are quantitative characteristic differences between pigmented and non-pigmented melanomas; however, further research characterizing pigment particle types and sizes may provide more information on establishing differences between pigmented BCC and melanomas. While the sensitivity and specificity of differentiating skin cancers from normal skin ranged from about 78% to 92% in our studies, additional data on each of these lesions is needed to be able to better differentiate between pigmented BCC and different melanocytic lesions. 

## 5. Conclusions

While both pigmented and non-pigmented melanomas have similar characteristics, including new 80, 130, and 250 Hz VOCT peaks, pigmented melanomas have larger 80 Hz and smaller 250 Hz peaks. The 80 and 250 Hz peaks can be used as quantitative characteristics to identify differences between different melanomas. While the sensitivity and specificity of differentiating skin cancers from normal skin range from about 78% to 92%, additional data on each of these lesions is needed to improve sensitivity and specificity to be able to differentiate pigmented BCC from other melanocytic lesions non-invasively. In addition, the use of AI, conducted on lesion histopathology, mechanovibrational data, and pigmented lesion light-scattering properties, may provide better identification and typing of melanocytic skin lesions. 

## Figures and Tables

**Table 1:** Statistical significance differences between resonant frequency peaks for normal skin, non-pigmented melanoma, and pigmented melanoma. The resonant frequency of normal cells (50 Hz), new cancer-associated cells (80 Hz), dermal collagen (100 Hz), new blood vessels (130 Hz), and new fibrotic tissue (250 Hz) are derived from the results of previous studies [1]. *p*-values in red are statistically different for pigmented and non-pigmented melanomas based on an unpaired one-tailed Student’s *t*-test.

	Normal Skin	Pigmented Melanoma	Non-Pigmented Melanoma
**50 Hz**			
**Normal Skin**	-	0.051	**0.00004**
**Pigmented Melanoma**		-	0.31
**80 Hz**			
**Normal Skin**	-	**3 × 10^−10^**	**2.9 × 10^−7^**
**Pigmented Melanoma**		-	**1.4 × 10^−5^**
**100 Hz**			
**Normal Skin**	-	0.17	**0.025**
**Pigmented Melanoma**		-	0.23
**130 Hz**			
**Normal Skin**	-	**0.003**	**6.2 × 10^−9^**
**Pigmented Melanoma**		-	0.1
**250 Hz**			
**Normal Skin**	-	0.46	**0.00002**
**Pigmented Melanoma**		-	**0.005**

**Table 2:** Thickness comparison between non-pigmented and pigmented melanomas measured using optical coherence tomography images and pixel intensity versus depth plots. *p*-values of statistical differences were calculated using an unpaired one-tailed Student’s *t*-test. Data shown indicates that pigmented melanomas scatter more light than non-pigmented melanomas, indicating that the melanin stacking in pigmented melanomas is different from that of pigmented melanomas. This is probably due to the tighter packing of the melanin pigment in pigmented melanomas.

	Average	SD
**Pigmented Melanoma**	256 μm	29.4 μm
**Non-Pigmented Melanoma**	277 μm	30.1 μm
*p*-value: 0.007		

**Table 3:** Results of machine learning to define the specificity and sensitivity of skin cancer diagnosis compared to normal skin.

	BCC	SCC	Melanoma
Sensitivity	90.9%	91.6%	83.33%
Specificity	87.50%	87.50%	77.77%

## Data Availability

Data contained in this study can be found at optovibronex.com (accessed on 27 February 2023).
